# Complete Genome Sequences of 10 Lactococcal *Skunavirus* Phages Isolated from Cheddar Cheese Whey Samples in Canada

**DOI:** 10.1128/MRA.00098-21

**Published:** 2021-04-15

**Authors:** Laurie Doré, Gabrielle Pageau, Françoise Bourque-Leblanc, Marie-Ève Dupuis, Roxanne Lessard-Hurtubise, Geneviève Lacasse, Pier-Luc Plante, Simon Labrie, Alice P. Jolicoeur, Geneviève M. Rousseau, Denise M. Tremblay, Sylvain Moineau

**Affiliations:** a Département de Biochimie, de Microbiologie et de Bio-informatique, Faculté des Sciences et de Génie, Université Laval, Québec, QC, Canada; b Groupe de Recherche en Écologie Buccale, Faculté de Médecine Dentaire, Université Laval, Québec, QC, Canada; c Centre de Recherche en Infectiologie de l’Université Laval, axe Maladies Infectieuses et Immunitaires, Centre de Recherche du CHU de Québec-Université Laval, Québec, QC, Canada; d SyntBioLab, Inc., Lévis, QC, Canada; e Félix d’Hérelle Reference Center for Bacterial Viruses, Université Laval, Québec, QC, Canada; Queens College CUNY

## Abstract

We report the complete genome sequences of 10 virulent phages of the *Skunavirus* genus (*Siphoviridae*) that infect Lactococcus lactis strains used for cheddar cheese production in Canada. Their linear genomes range from 28,969 bp to 31,042 bp with GC contents of 34.1 to 35.1% and 55 to 60 predicted open reading frames (ORFs).

## ANNOUNCEMENT

Lactococcus lactis strains are added to milk to manufacture a wide variety of cheeses worldwide. The most common cause of slow milk fermentation, which leads to low-quality fermented products, is virulent phages infecting these strains (1). Lactococcal phages are classified into several groups (2), with phages belonging to the *Skunavirus* genus (formerly 936) being the most common (3). Constant phage monitoring in dairy factories is essential for adapting antiphage measures and preventing fermentation failure (4). Here, we report the genomic characterization of 10 new virulent phages (FB3, FB6, FB10, FB14, GL7, GP13, GP14, GP15, RH6, and RH10) of the *Skunavirus* genus. Phages were isolated from 2007 to 2019 from whey samples obtained from a Canadian cheddar cheese factory.

L. lactis strains were grown at 30°C in M17 medium with 0.5% (wt/vol) lactose (LM17). Phages were isolated using the double-layer plaque assay (5) on LM17 medium supplemented with 10 mM CaCl_2_. Phage genomic DNA was extracted using phenol-chloroform (6) from high-titer (>10^9^ PFU/ml) filtered (0.45-μm filter) lysates. Sequencing libraries were prepared using a Nextera XT DNA library preparation kit and sequenced with Illumina MiSeq (250-nucleotide paired-end reads). Reads were cleaned using Trimmomatic v0.36 (7) and assembled to obtain circular complete sequences using Ray v3.0.1 (8) with k-mer sizes of 21, 31, 41, 51, 71, and 91 and SPAdes v3.13 (9). Open reading frames were predicted using GeneMark (prokaryotic) v3.25 (10), the PECAAN annotation tool (https://discover.kbrinsgd.org/autoannotate/), and Geneious v11.1.5 (11), with the following principles: genes started with ATG, GTG, or TTG codons and were preceded by a Shine-Dalgarno sequence similar to 5′-AGAAAGGAGGT-3′ (12). Coding sequences of 30 or more amino acids were annotated, and deduced proteins were searched for function using BLAST v2.10.0 and a cutoff E value of 0.001. We searched tRNAs with ARAGORN v1.2.38 (13) and tRNAscan-SE 2.0 (14). Annotations were also manually curated by comparing them with other *Skunavirus* genomes. Unless defined, default parameters were used for all software.

The genome size (from 28,969 to 31,042 bp), number of predicted open reading frames (ORFs) (55 to 60), and GC content (34.1 to 35.1%) for each phage are reported in Table 1. The percentage of deduced proteins with an assigned function ranged from 32.1 to 39.0%. *Cos* sites were found in the 10 phages by sequence homology and were identical (5′-CACAAAGGACT-3′) to other *Skunavirus* phages (15). The average nucleotide identities (ANI) were calculated with a BLAST+ analysis in JSpeciesWS v3.7.3 (16) and are listed in Table 1.

**TABLE 1:** Characteristics and accession numbers of the 10 lactococcal *Skunavirus* phages

Phage name	Isolation mo-yr	L. lactis host strain	Genome length (bp)	No. of ORFs	GC content (%)	GenBank/SRA accession no.	No. of reads	Most closely related phage(s)	ANI (%)
GL7	12-2007	SMQ-747	29,705	60	34.3	MW041638/SRR13677537	295,756	LP0004a (Germany)	92.1
FB3	4-2018	SMQ-1021	28,969	56	34.8	MW041632/SRR13677536	383,226	FB6^*a*^	94.3
								PhiF.17 (Netherlands)	92.4
FB6	7-2018	SMQ-1021	30,766	59	34.5	MW041633/SRR13677535	177,640	PhiF.17 (Netherlands)	93.7
RH10	9-2018	SMQ-491	30,860	56	34.1	MW041636/SRR13677534	367,860	P4565 (NA)^*b*^	95.1
FB10	10-2018	SMQ-746	30,272	55	35.1	MW041640/SRR13677533	365,878	RH6^*a*^	99.9
								CB19 (Canada)	92.5
RH6	10-2018	SMQ-746	30,299	55	35.1	MW041639/SRR13677532	453,427	CB19 (Canada)	92.4
FB14	12-2018	SMQ-1021	31,042	60	34.5	MW032477/SRR13677531	130,824	FB3^*a*^	96.0
								FB6^*a*^	95.6
								CaseusJM1 (Ireland)	93.7
GP14	3-2019	SMQ-999	29,200	55	34.6	MW041634/SRR13677530	207,995	GP15^*a*^	99.5
								jm2 (Ireland)	94.1
GP13	4-2019	SMQ-1420	29,126	55	34.9	MW041637/SRR13677529	222,828	CHPC964 (USA)	93.7
GP15	4-2019	SMQ-999	29,166	55	34.6	MW041635/SRR13677528	249,273	jm2 (Ireland)	94.0

a This study.

b NA, no country available.

Phages FB10, GL7, GP13, GP14, GP15, RH6, and RH10 possess an early-expressed gene that codes for a methyltransferase. These methylases likely protect the viral genome against a specific host endonuclease during intracellular replication. Phage FB14 also carries a methyltransferase-coding gene, but it is located in the late-expressed region. This methylase may perform regulatory functions (17, 18). Six genomes contained tRNA-Pro and tRNA-Trp. Phages GP13 and RH10 had only tRNA-Pro, and phages GP14 and GP15 did not carry any tRNA.

### Data availability.

The phages are available at www.phage.ulaval.ca. The genome sequences and raw data are available under the GenBank and SRA accession numbers reported in Table 1.
